# Evaluation of Sensitization Program on Occupational Health Hazards for Nursing and Allied Health Care Workers in a Tertiary Health Care Setting

**DOI:** 10.3389/fpubh.2021.669179

**Published:** 2021-06-16

**Authors:** Manisha Naithani, Meenakshi Khapre, Rajesh Kathrotia, Puneet Kumar Gupta, Vandana Kumar Dhingra, Shalinee Rao

**Affiliations:** ^1^Department of Biochemistry, AIIMS Rishikesh, Uttarakhand, India; ^2^Department of Community and Family Medicine, AIIMS Rishikesh, Uttarakhand, India; ^3^Department of Physiology, AIIMS Rajkot, Gujarat, India; ^4^Department of Microbiology, AIIMS Bilaspur, Himachal Pradesh, India; ^5^Department of Nuclear Medicine, AIIMS Rishikesh, Uttarakhand, India; ^6^Department of Pathology and Advanced Center of Continuous Professional Development, AIIMS Rishikesh, Uttarakhand, India

**Keywords:** continuous professional development (CPD), effectiveness, healthcare workers (HCW), occupational health hazards, sensitization, training, vaccination

## Abstract

**Background:** Occupational health hazard pertaining to health care providers is one of the neglected areas that need serious attention. Any compromise in their safety would result in reduction in workforce, which may affect patient care, keeping in mind the wide gap between the required number and actual health care workers (HCWs) available in the world over.

**Aim:** This study was undertaken to evaluate the change in knowledge through a sensitization training program on occupational health hazards and vaccination for HCWs.

**Materials and Methods:** Participants of the study included nursing and allied HCWs of a tertiary care health institute in Uttarakhand, India. Multiple training sessions, each of around 180 min, were held periodically in small groups with 20–40 participants over 2 years. Participants were assessed with pretest and posttest questionnaires, and feedback was taken. Questionnaires comprised three categories: general safety and ergonomics, biological hazards, and chemical and radiation hazards. Data of incident reporting for needlestick injury from 2017 to 2019 were retrieved. All data were compiled in Excel sheet and analyzed.

**Results:** A total of 352 participants were included in the study. Mean ± SD for pretest and posttest scores were 5.3 ± 2.13 and 11.22 ± 2.15, respectively. There was considerable improvement in knowledge, which was found to be statistically significant with *p*-value of 0.001 for all categories. Participants in their feedback suggested for inclusion of psychosocial aspect in further training programs.

**Conclusion:** Low baseline knowledge prior to attending the course highlights a need for an intervention through such structured sensitization program to create awareness and educate HCWs on common occupational health hazards and vaccination. Statistically significant improvement in posttest knowledge highlights effectiveness of the training program. A drastic rise in incident reporting for needlestick injury reflects fairly good impact of training program. Regular and appropriate form of training can reduce injuries resulting from occupational hazards and ensure healthy workforce contributing toward a positive impact on national economy.

## Introduction

The World Health Organization (WHO) has described occupational health as development, promotion, protection, enhancement, and enabling of all aspects, i.e., physical, mental, and social health of workers (1). All work environments, including the health care sector, pose various hazards for the workers. Health care workers (HCWs) include professional medical workers such as doctors, nurses, and all paramedical workers, assistants, students, or trainees and general staff. WHO has described various occupational hazards in the workplace such as air contaminants, chemical hazards, radiation hazards, biological hazards, physical hazards such as noise and heat, ergonomic hazards, and psychosocial hazards (1).

There are many resources available to develop programs for addressing occupational health hazards, and every organization may design its own program as per availability of professionals and other resources. However, sensitization of all involved personnel is the first step toward a healthy work environment. One of the primary reasons for occupational injuries in HCWs is not being aware of the hazards that they are exposed to in their workplace. Inadequacy of knowledge or non-adherence to the safety preventive measures leads to chronic illnesses, functional impairment, disabilities, and even sometimes death, which affects the individual's family, institute, and manpower resource of the country. An assessment of baseline knowledge of HCWs will be useful to determine their existing understanding so that interventions can be planned through focused training programs for their enlightenment and update (2). Regular appropriate training can reduce injuries resulting from occupational hazards, which in turn may have a positive impact on national economy especially in developing or low-income countries (3).

## AIM

This study was undertaken to assess the baseline knowledge of HCWs and the immediate effect of sensitization training program on occupational hazards through objective assessment.

## Materials and Methods

**Figure 1:**
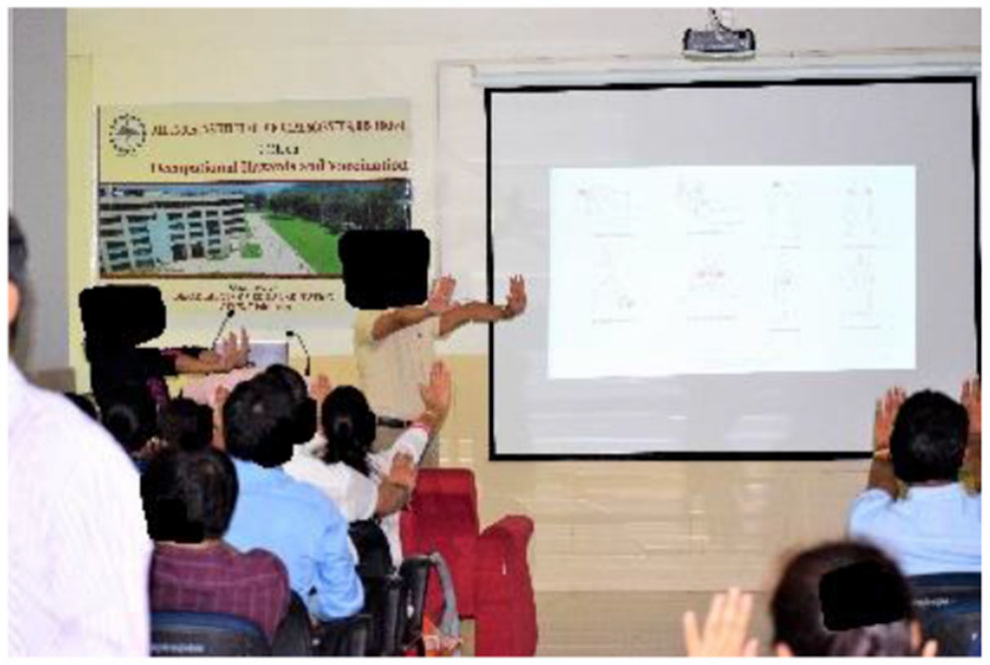
Participants performing exercise while being demonstrated by an instructor during delivery of ergonomic training module.

This was a study done at a tertiary care medical institute in Uttarakhand, India, which included data from 2018–2020. Participants of this study included nursing and allied HCWs (technicians). A structured training module on occupational hazards was designed for sensitizing and educating the participants. Basic information about vaccination for HCWs was also covered as a component of protection from biological hazards. This initiative was a part of the various regular training programs scheduled for continuous professional development program. The training workshop comprised an interactive session of 3-h duration, which included general protocols, biological, chemical, radiation, and ergonomic health hazards. Session submodules consisted of short lectures using PowerPoint presentation with case scenarios followed by interactive discussion. One session each was conducted on general protocols of safety in hospital sector and biological hazards and vaccination of 30-min duration, followed by chemical hazards for 30 min, ergonomic hazards for 15 min (Figure 1), and radiation safety 20 min. Social and psychosocial factors were not included in our program. Relevant resource materials from standard guidelines, such as WHO, Centers for Disease Control and Prevention (CDC), the Occupational Safety and Health Act of 1970 (OSH Act), and Atomic Energy Regulatory Board (AERB), were utilized (1, 4–6). The sessions were conducted in small groups with maximum 40 participants. Nursing and allied HCWs participated in the training. Evaluation of the effect of intervention was done using objective assessment (posttest) through the same set of questionnaires as used in the pretest. The pretest was conducted to assess the baseline knowledge of participants with multiple-choice questions prior to starting the training program. The questionnaire consisted of 15 questions within three categories, i.e., general safety and ergonomics (six questions), biological hazards (four questions), and chemical and radiation hazards (five questions). All questionnaires were evaluated for response. Each category was analyzed separately, and results were assessed; i.e., mean, standard deviation, and test of significance were applied using SPSS software version 23. Feedback was taken from participants with close-ended and open-ended questions and was further analyzed. On completion of the training program with a score of 80% and greater, certificates were distributed to participants. Resource faculty were also provided with appreciation certificate.

Data of needlestick injury (NSI) including blood and body exposure reported by HCWs at the institute was collected and analysed.

## Results

A total of 351 participants were trained over 12 sessions, which included 324 nursing officers of different cadres, and 27 laboratory technicians. Participants were predominantly females (254; 72.4%), and the rest were males (97; 27.6%). Pretest and posttest performances were analyzed with mean ± SD pretest and posttest scores of 5.3 ± 2.13 and 11.22 ± 2.15, respectively. Statistically significant improvement in knowledge was noted in posttest for all submodules (Table 1). Maximum improvement in knowledge was noted for a session on biological hazards (Table 1).

**Table 1:** Category-wise pretest and posttest scores and total scores (*n* = 351).

**Category**	**% Score in pretest**	**% Score in posttest**	**Difference in pretest vs. posttest**	***p*-value**
General safety and ergonomics	47	88	41%	0.001
Biological hazards	27	75	48%	0.001
Chemical and radiation hazards	39	74	35%	0.001

### Feedback on Training Module

**Figure 2:**
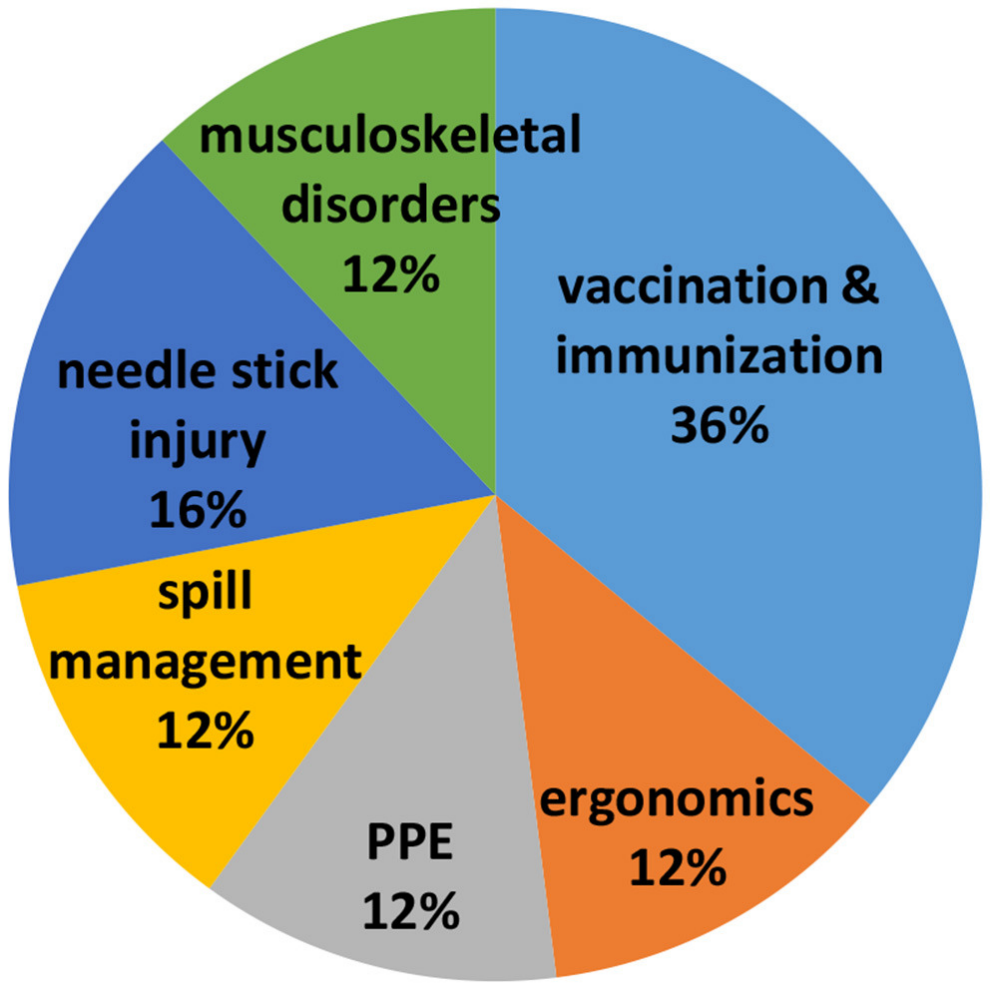
Usefulness of content as per participants' feedback.

On analysis of participants' perception about workshop and key learning points learned, it was revealed that all participants felt time accorded to training was adequate (100%). The content of program was excellent (50.8%) to good (48.2%) for the majority, whereas delivery of content was equally excellent (45.6%) to good (52.4%). Method used for training was considered appropriate (100%). All participants (100%) responded that there was skill enhancement after training and considered training content very useful. Participants responded that biological hazards and vaccination submodules were highly useful (Figure 2). Participants recommended few suggestions for future training workshops (Table 2).

**Table 2:** Feedback of participants.

**Sessions that need more elaboration**	**Any other topics that can be included in this workshop**	**Take-home messages(as per participants)**
Biological hazards, 35% Chemical hazards, 30% Psychosocial hazards, 22% Vaccination, 5% Immunization, 5% Spill management, 3% Safe disposal and handling of cytotoxic drugs, 5%	•Respiratory safety •Ergonomic specific to nursing •Brachytherapy •Water hygiene, food hygiene, nutrition	•Prevent musculoskeletal disease with appropriate posture •Get vaccinated as soon as possible •Self-protection methods •Prevention is better than cure •Minimize radiation exposure •Follow proper use of personnel protective equipment and their disposal, prevention from needlestick injury •Occupational hazards can be prevented to great extent by proper training and management •Follow ergonomics—maintain good body (posture to avoid future disability)

A considerable increase in reporting of NSI including blood and body exposure cases was noted (Table 3).

**Table 3:** Needlestick injury including blood and body exposure cases reported at the institute from 2017 to 2019.

**Time frame**	**Total cases reported**	**Among nursing officers**
2017 (From September to December)	10	2
2018 (From January to December)	91	45
2019 (From January to September)	99	42

## Discussion

The health care sector is a field for improving the health of others; however, ironically, it possesses every kind of health hazards and has been found to have a higher-than-average rates of occupational health hazards (7). While the HCWs focus on providing patient care, they become exposed to many occupational hazards that could affect their health and well-being (8). The most common occupational hazards found in the health care setting are chemical, biological, physical, and psychosocial hazards; however, many workers are unaware of the hazards they are exposed to, and this may prove to be injurious for themselves and their colleagues, as well as the patients (9–11).

The hazards for medical professionals have been defined broadly in older documents; however, currently, agencies such as the CDC, the National Institute for Occupational Safety and Health, and the Occupational Safety and Health Administration (OSHA) provide guidelines and standards for information and prevention on occupational hazards for all including medical workers (4–6, 12). These are acceptable as standard guidelines in world over for practical purpose. Often, HCWs, especially in the developing world, are aware of the hazards but may be unable to help themselves from their risks due to knowledge about the same or lack of resources (8). A study conducted by Aluko et al. (8) highlighted that HCWs hardly practiced safety measures at the workplace despite having a good level of knowledge about the preventive means for occupational hazards.

A review published by Rai et al. (13) documents that practice of occupational hazard risk reduction strategies is deficient. Rai et al. documented that most of the studies are focused on biological hazards, whereas, research studies on other hazards were limited in comparison. Our training program comprehensively covered general policies and measures, biological, chemical, musculoskeletal, and radiation occupational hazards and its preventive measures.

It is a well-known fact that there is a vast deficiency of HCWs all over the world. Most of escalated health care costs are attributed to newer equipment and drugs while escalation of expenses on nursing, or manpower development and maintenance are negligible. These have led to poorer patient care outcomes. These have also led to negative outcomes among nursing and other HCWs such as higher absenteeism or earlier retirement. Issues such as musculoskeletal disorders, stress, or dermatological issues and many more directly or indirectly associated with their occupation are responsible for this situation even in the developed countries (14). Therefore, to safeguard the health of HCWs in low- to middle-income countries, authorities should consider and prioritize this as one of the public health issues. Focused research to assess knowledge on occupational hazards will give an insight into lacunae and gap in knowledge of HCWs.

It has been noted that in comparison to other workers, such as miners, the occupational health of HCWs fades in the background as they are at the helm of caring for the vulnerable sick and injured. Partial knowledge may be dangerous, especially in the health care sector where HCWs are at the interface of disease and health of the people. Although, HCWs have better knowledge about occupational hazards compared to the other sectors such as sawmill workers, farmers, or miners, more than a third of HCWs failed to recognize work-related health hazards (8, 15).

NSIs are the commonest accidents among HCWs followed by direct contact with blood, chemical burns, and floor injuries such as slipping. In our program, the session on biological hazards and vaccination was scored as the most useful as per participant feedback. Our study also showed a positive trend in reporting of NSI, which reflects definite impact of the training program in this group of HCWs. Abuduxike et al. in their study on HCWs evaluated experience of NSIs and factors related to it through self-administered questionnaire and found low adherence to standard precautions. They further suggested such behavior and practice can be changed through a regular, focused training program based on occupational risk, and exposure (16).

The major hazardous activities in a health care setting have been known to be injection, cleaning, patient care, bedding, dressing of wounds, medication, and surgical operation; all of these activities involve biological hazard exposure (17). This could be attributed to the fact that despite their primary training in handling infectious materials, there are maximum reported incidents of direct skin contact with infectious materials and NSIs among HCWs. This is an indicator of training reinforcement to bring about behavioral changes among HCWs and improved practices (18).

There are not many published reports pertaining to occupational health care among the HCWs from the Indian subcontinent. Thus, it may be assumed that not many institutions/health care facilities are performing such a program in this setting (19). The Advanced Center of Continuous Professional Development at our institute took over this task and developed this program for initiating health promotion. Training modules were developed by the resource persons who were specialized in the respective areas of the module allotted to them. There were four modules in each session, including major occupational hazards such as general policies and protocols, biological (bloodborne pathogens, tuberculosis), ergonomic (musculoskeletal problems), and radiation hazards and chemical hazards (toxic chemicals in the laboratory, latex allergy, and chemotherapeutic drugs). The participants responded in their feedback that they were highly satisfied by the training module. As per the OSHA guidelines, organizations must involve workers in the program and accommodate training and all actions pertaining to occupational safety within working hours. We complied with these guidelines in our study (5). Specialized occupational health professionals usually address these issues; however, these trained professionals may not be available everywhere even in the developed countries and even if they may not able to cater to the vast number of health care professionals (1). In such situations, HCWs may themselves take up the additional responsibility of health promotion at the workplace.

There was a statistically significant improvement in pretest and posttest scores among all participants for the workshop. Sensitization to the various aspects of occupational hazards was thus achieved in our workshop. The salient feature of the present study was evidence-based information on baseline knowledge of HCWs on workplace health hazards and preventive/safety measures. The study reveals necessity to develop and implement strategies including focused training to improve the knowledge, practice, and compliance of preventive measures against occupational health hazards (16). One of the factors for success is the number of participants in the program. Our sessions were limited to <40 participants. This has been shown to be an important factor as larger-sized batches (60–80) have shown to have an adverse effect on training/interaction in other studies (19). This program is an ongoing continuous feature in our institution, and it is ensured that every nursing staff/student and paramedical worker is mandatorily trained/sensitized.

Analysis of feedback from participants in our training revealed that time accorded to training was adequate, content and delivery of content were found to be acceptable, and they felt that the session was useful for them. The participants recommended including a session on stress and psychosocial hazards, which was lacking in our training module. Myths or fears can cause wrong practices/avoidance of certain work practices; on the other hand, overconfidence due to ignorance may lead to unnecessary injury or exposure to hazards. It has also been seen in the past that HCWs are aware of their lack of knowledge and information about occupational hazards and realize the need for training and awareness (20).

Stress and psychosocial hazards are pertinent problems in any health care setting. Rosenberg et al. conducted a questionnaire-based study on Finnish anesthetists and found that a higher number of abortions were noted among anesthetists, as compared to incidence before entering anesthetic work. The possible reasons as put forward by them included anesthetic gases, smoking, and psychosocial hazard (21). Their study also revealed that gestation time for full-term pregnancies and miscarriages was shorter in the anesthetist group as compared to the pediatrician group (21). Regular appropriate training can reduce injuries resulting from occupational hazards, which in turn may have a positive impact on national economy (2).

Routine training and reinforcement programs based on accepted guidelines on safety practices through mock drills in all health facility centers should be made mandatory (22). Sessions resource could be posted online for web-based learning, and this mode may prove effective for delivery of knowledge as noted in a study conducted by Tung et al. (23). A recent observational study by Cattelan et al. documented the positive impact of an effective training program in preventing infection with severe acute respiratory syndrome coronavirus 2 infection in HCWs. This reiterates the role of quality training program for preventing biological hazard from acquiring highly infectious disease (24).

Positive reinforcement of employees can be performed by the ways of incentives or recognition. Certificates of participation were awarded to all the participants, and certificates to resource faculty as well in our program.

## Limitations

It has been seen that psychosocial factors and various other stressors have an impact on HCWs, especially nurses (25). We did not cover psychosocial hazards; however, it will be included in future sessions. There is a need to include assessment of active reporting about safety and health concerns by employees; however, this record was not available for analysis.

## Conclusion

To, the best of our knowledge, this is one of the first documented studies in India, where basic comprehensive sensitization training program on occupational health hazard training has been performed and evaluated. Low baseline knowledge prior to attending the course, highlights a need for an intervention through such structured sensitization program to create awareness and educate HCWs on common occupational health hazards and vaccination. Statistically significant improvement in posttest knowledge highlights the effectiveness of the training program. Regular and appropriate training can reduce injuries resulting from occupational hazards and ensure healthy workforce contributing toward a positive impact on national economy.

## Data Availability Statement

The original contributions presented in the study are included in the article/Supplementary Material, further inquiries can be directed to the corresponding author/s.

## Ethics Statement

The studies involving human participants were reviewed and approved by AIIMS/IEC/2020/788-dated 21/11/2020.

## Author Contributions

MN, VD, and SR: conceptualization and manuscript preparation and writing. MK and PG: assistance in data collection. RK: statistical analysis. SR: overall supervision, data collection, and editing manuscript. All authors contributed to the final article and approved the submitted version.

## Conflict of Interest

The authors declare that the research was conducted in the absence of any commercial or financial relationships that could be construed as a potential conflict of interest.
