# Supplementary material for: Evaluation of Sensitization Program on Occupational Health Hazards for Nursing and Allied Health Care Workers in a Tertiary Health Care Setting
Source: Front Public Health. 2021 Jun 16;9:669179. doi: 10.3389/fpubh.2021.669179 (PMC8248788; doi:10.3389/fpubh.2021.669179)
Supplement: Supplementary file 1 [file Data_Sheet_1.docx]

| **Designation** | **pre test score** | **post test score** | **P-value** |
| --- | --- | --- | --- |
| Nursing Officer | 5.3±2.13 | 11.22±2.15 | <0.01 |
| Technician& interns | 3.4±1.42 | 8.42±2.22 | <0.01 |

**Overall Results: Table 1**

|  | **Nursing Officer** | **N=324** |  |  |
| --- | --- | --- | --- | --- |
|  | **% score in pre test** | **% score in post test** | **difference of pre vs post test % score** | **P-value** |
| Q1- Which one of the following is correct regarding risk of transmission following occupational exposure? | 35% | 95% | 60% | 0.001 |
| Q2- Identify the hierarchy of controls that should be implemented to control hazards in the workplace. | 9% | 89% | 80% | 0.001 |
| Q3- The following symbol refers to: | 78% | 91% | 13% | 0.001 |
| Q4- IfHBsAbtitre falls down <10mIU/ml for a previously protected HCW and if he gets NSI from source positive/ unknown exposures: what to do according to CDC recommendation? | 12% | 88% | 76% | 0.001 |
| Q5- What is the most common site for pain due to ergonomical hazards | 38% | 80% | 42% | 0.001 |
| Q6: What is the most important factors to consider for reduce exposure to radiation? | 63% | 83% | 19% | 0.001 |
| Q7- A HCW coming to you saying that he has been vaccinated with two doses of Hepatitis B vaccine 3 years back but no documentation. He wants to know how should he continue up with the vaccination. Suggest the appropriate vaccination strategy, according to CDC recommendation? | 31% | 83% | 52% | 0.001 |
| Q8- What does the colour code blue stand for | 61% | 95% | 35% | 0.001 |
| Q9- Main Principle for radioactive waste disposal is: | 28% | 82% | 53% | 0.001 |
| Q10- Correct sequence of donning PPE is: | 47% | 75% | 28% | 0.001 |
| Q11- “Double person Double lock” to use for storage of | 66% | 96% | 29% | 0.001 |
| Q12- The following equipment is used for monitoring: | 35% | 47% | 12% | 0.001 |
| Q13- A blood is spilled on the floor of approximate 4 cm radius now what to do? | 19% | 55% | 35% | 0.001 |
| Q14- What is annual limit of effective dose for Public? | 6% | 65% | 58% | 0.001 |
| **Total Score (Mean ± SD)** | 5.3±2.13 | 11.22±2.15 |  | 0.001 |

**Nursing Officers -Pre and Post test Results Item/Question wise : Table 2**

|  | **Technicians** | **N=27** |  |  |
| --- | --- | --- | --- | --- |
|  | **% score in pre test** | **% score in post test** | **difference of pre vs post test % score** | **P-value** |
| Q1- Which one of the following is correct regarding risk of transmission following occupational exposure? | 39% | 75% | 36% | 0.001 |
| Q2- Identify the hierarchy of controls that should be implemented to control hazards in the workplace. | 18% | 75% | 57% | 0.001 |
| Q3- The following symbol refers to: | 63% | 75% | 12% | 0.001 |
| Q4- If HBsAbtitre falls down <10mIU/ml for a previously protected HCW and if he gets NSI from source positive/ unknown exposures: what to do according to CDC recommendation? | 18% | 94% | 76% | 0.001 |
| Q5- What is the most common site for pain due to ergonomical hazards | 54% | 75% | 20% | 0.001 |
| Q6: What is the most important factors to consider for reduce exposure to radiation? | 59% | 75% | 16% | 0.001 |
| Q7- A HCW coming to you saying that he has been vaccinated with two doses of Hepatitis B vaccine 3 years back but no documentation. He wants to know how should he continue up with the vaccination. Suggest the appropriate vaccination strategy, according to CDC recommendation? | 25% | 77% | 52% | 0.32 |
| Q8- What does the colour code blue stand for | 30% | 77% | 47% | 0.9 |
| Q9- Main Principle for radioactive waste disposal is: | 28% | 73% | 46% | 0.9 |
| Q10- Correct sequence of donning PPE is: | 34% | 90% | 57% | 0.11 |
| Q11- “Double person Double lock” to use for storage of | 18% | 76% | 58% | 0.001 |
| Q12- The following equipment is used for monitoring: | 51% | 72% | 22% | 0.001 |
| Q13- A blood is spilled on the floor of approximate 4 cm radius now what to do? | 23% | 65% | 42% | 0.001 |
| Q14- What is annual limit of effective dose for Public? | 18% | 75% | 57% | 0.04 |
| **Total Score (Mean ± SD)** | 3.4±1.42 | 8.42±2.22 |  | 0.001 |

**Technicians- Pre and Post test Results Item/Question wise : Table 3**

**Usefulness of Sessions as per feedback : Fig 1**

|  | **No of participants** | **Post test scores** |
| --- | --- | --- |
| Session 1 | 36 | 10.98 |
| Session 2 | 30 | 10.5 |
| Session 3 | 34 | 11.02 |
| Session 4 | 15 | 8.42 |
| Session 5 | 26 | 10.5 |
| Session 6 | 40 | 11.03 |
| Session 7 | 34 | 9.88 |
| Session 8 | 22 | 10.97 |
| Session 9 | 23 | 11.2 |
| Session 10 | 38 | 9.32 |
| Session 11 | 31 | 10.58 |
| Session 12 | 22 | 11.32 |
|  | Total=351 |  |

**Table 4: Effect on Number of participants on session**

| **Sessions which need more elaboration** | **Any other topics, which can be included in this workshop?** | **Take home messages**  **(as per participants)** |
| --- | --- | --- |
| Biohazard - 35%  Chemical hazard -30%  Psychsocial hazard -22%  Vaccination-5%  Immunization -5%  Spill management-3% | - Safe disposal and handling of Cytotoxic drugs - Respiratory safety - Ergonomic specific to nursing - PPE in OT/ward - CPR - Teletherapy, brachytherapy - water hygiene, food hygiene nutrition | - Prevent MSD with appropriate posture - Get vaccinated as sooner possible - Self-Projection Methods - Vaccination when Needed - prevention is better than cure - Minimize radiation exposure - Following of proper use of personnel protective equipment & their disposal, prevention from needle stick injury - Occupational hazards can be prevented to great extent by proper training and management - Follow Ergonomics -> Maintain good body (posture to avoid future disability) |

**Results of Feedback : Table 5**
